# Acute Hepatitis A and Hepatitis B Coinfection in a Young Female: A Case Report and Literature Review

**DOI:** 10.1155/2023/1809020

**Published:** 2023-06-03

**Authors:** Narges Lashkarbolouk, Behnaz Khodabakhshi, Mahdi Mazandarani

**Affiliations:** ^1^Endocrinology and Metabolism Research Center, Tehran University of Medical Sciences, Tehran, Iran; ^2^Infectious Disease Research Center, Golestan University of Medical Sciences, Gorgan, Iran

## Abstract

**Background:**

Hepatitis is one of the common infectious diseases that can infect patients in various forms. Based on their characteristics and clinical features, they can cause irreparable complications in patients. Coinfections and superinfections between its variant have been reported, but the coinfection of acute HAV and HBV is rarely reported. *Case Presentation*. In this case report, we presented a case with severe malaise, nausea, vomiting, generalized jaundice, and a history of recent tattooing and travel to the HAV endemic area. In our evaluation, she had a positive HBsAg, HBeAg, anti-HBs IgM, anti-HAV IgM, and negative result in HCV antibody, HIV antibody, and anti-HAV IgG. The coinfection of HAV/HBV was confirmed for her.

**Conclusion:**

Physicians should differentiate hepatitis A and hepatitis B superinfection or coinfection, based on history and laboratory testing, to prevent complications with appropriate treatment.

## 1. Introduction

Hepatitis is an infectious disease that primarily affects the liver; depending on the nature of the virus, it can range from mild and self-limiting acute infection to fulminant cases. Hepatitis is almost always caused by hepatitis A, B, C, D, and E viruses, and except for the hepatitis B virus (HBV), which has a DNA-based genome, all of these viruses are of the RNA genome type. Hepatitis B virus (HBV) and hepatitis C virus (HCV) cause both acute and chronic infections, whereas hepatitis A, E, and D cause only acute disease. The most common causes of fulminant hepatitis are HAV and HEV infection. In 2015, viral hepatitis caused 1.34 million deaths worldwide, as well as tuberculosis (1.37 million), HIV infection (1.06 million), and malaria (0.44 million). Moreover, chronic hepatitis complications accounted for 96% of hepatitis-related deaths in 2015 [1, 2].

Hepatitis A virus (HAV) is one of the most prevalent causes of acute viral hepatitis worldwide and is transmitted through the fecal-oral route. The symptoms of this virus are self-limiting, but it can rarely cause liver failure and death. The exposure risk factors include contaminated water and food, contact with an infected individual, unhygienic living conditions, intravenous drug users, homelessness, and incarceration. Due to its high sensitivity and specificity, the presence of anti-hepatitis A IgM in the HAV test can clarify the diagnosis of HAV [3, 4].

HBV causes acute and chronic hepatitis infections, fibrosis, cirrhosis, and hepatocellular carcinoma. Using an enzyme immunoassay, the presence of hepatitis B surface antigen (HBsAg) in serum or plasma can validate the diagnosis of acute infection. HBV is transmitted through contact with infected blood or body fluids through the skin or mucosa. Important transmission routes include perinatal transmission, sexual transmission, homosexual intercourse in men, and injection transmission [5, 6].

The prevalence of HBV and HAV has changed in recent decades due to systematic vaccination programs, diagnostic efforts, and effective treatment. Nevertheless, HBV and HAV are significant public health problems with a high mortality rate. In 10–15% of HBV infections, acute HBV/HCV coinfection and HAV superimposed on chronic HBV have been mentioned and reported. Acute HBV/HAV coinfection is rare and less frequently reported [7, 8].

This study investigated HBV/HAV coinfection in a young female with jaundice and myalgia as primary symptoms.

## 2. Case Presentation

A 24-year-old female was admitted to our hospital with malaise and jaundice as chief complaints. Ten days before hospitalization, the patient experienced nausea, vomiting, anorexia, malaise, and myalgia. One week after the onset of symptoms, she experienced fever, chills, and yellow-brown stools and developed dark urine, and jaundice became more severe. Throughout the course of the illness, the patient did not experience headaches, photophobia, pharyngitis, or coryza symptoms. Moreover, the patient did not report any weight loss, abdominal pain, changes in smell or taste, or itching.

The individual's lips were tattooed two months before admission. Furthermore, twenty days before admission, the patient traveled to Mashhad and reported acute nausea and vomiting that lasted three days. The patient had no history of drug or substance abuse, homelessness, multiple sexual partners, incarceration, or previous viral hepatitis infections; she tested negative for HBV and HIV infection 11 months before admission. The patient's immunization was carried out according to the national vaccination protocol, but the patient was not vaccinated against hepatitis A and B.

On physical examination, the vital signs were normal, scleral were icterus, and general jaundice was notable. The abdominal examination was negative for hepatosplenomegaly, asterixis, cutaneous stigmata, adenopathy, and edema. Due to the patient's condition and the risk of disease transmission, she was admitted to the isolation room.

Significant leucocytosis, anemia, aspartate aminotransferase (AST) level elevated to 2600 U/L, alanine aminotransferase (ALT) level elevated to 3590 U/L, alkaline phosphatase (ALP): 432 U/L, total bilirubin (TB): 15.8 mg/dL, and direct bilirubin (DB): 9 mg/dL were observed following laboratory evaluation. Her hepatitis test results were positive for HAV IgM, negative for HAV IgG, positive for HBV core IgM, and positive for HBsAg and HBeAg. However, human immunodeficiency virus- (HIV-), HDV-, and HCV-antibody testing were negative. The total protein and albumin levels in the serum were 5.7 g/dl and 3.2 g/dl, respectively. The partial thromboplastin time (PTT) and prothrombin time (PT) measurements were 41 and 18 seconds, respectively, and the international normalized ratio (INR) was 1.51 (Table 1).

Abdominal ultrasound was reported normal, with no biliary dilatation and extrahepatic obstruction. The coinfection of HBV and HAV was confirmed based on clinical examination and laboratory results. Due to her severe condition, tenofovir was administered as a supportive and antiviral treatment throughout therapy. After three days of treatment, her laboratory tests and symptoms began to improve, and within one week, she was discharged and advised to return for follow-up care. After two weeks of treatment with antiviral medication (tenofovir), her laboratory test was within normal range, and her clinical symptoms were completely resolved. Therefore, we discontinued antiviral treatment, and within one month of follow-up, the patient still did not have any clinical symptoms, and her laboratory tests were still within the normal range (Table 1).

## 3. Discussion

Hepatitis remains one of the most significant global health concerns. Even though hepatitis A and B are vaccine-preventable diseases, recent studies in the United States indicate an increase in the incidence of HAV infection between 2016 and 2018, with admission and mortality rates of 67% and 2.7%, respectively. In addition, HBV estimated acute infections remained stable from 2016 to 2019; it was 20,700 patients in 2019, and the prevalence of chronic HBV infection was estimated at 1.59 million patients in the same year [1–3, 7].

As stated previously, HAV is transmitted via the fecal-oral route. According to Miri et al. [9], Iran has a high prevalence of HAV infection and is considered an endemic country in the region. Our patient had previously traveled to Mashhad, Razavi Khorasan province, in northern Iran, and she developed symptoms during her trip, which lasted for three days [4–6, 9–11].

HBV, on the other hand, is transmitted through contact with a patient's blood and body fluids. In most cases, HBV is transmitted through sexual contact, drug injection equipment, and from the mother during delivery. Several diseases, such as HBV, HCV, and HIV, are also considered to be transmitted through tattooing; this is primarily due to sharing needles and other equipment and poor disinfecting. Our patient had a history of tattooing about two months before admission [9–12].

In patients with acute hepatitis, the spectrum of clinical symptoms can range from self-limiting in mild cases to fulminant hepatitis in severe cases. In most cases, patients typically develop anorexia, nausea, vomiting, arthralgia, myalgia, headache, photophobia, fever, and generalized icterus. Several of these symptoms were observed in our patients, where initial symptoms were nausea, vomiting, and anorexia, followed by malaise, myalgia, fever, chills, yellow-brown stools, dark urine, and jaundice.

IgM HAV is a serological hallmark test used to diagnose acute HAV infection in suspected patients. Positive autoimmune antibodies, such as rheumatoid factor (RF), can result in a false-positive IgM HAV test result. Negative anti-HAV IgG or negative HAV RNA confirms the false-positive condition in this instance [1–3, 5, 6].

Serum HBV virological markers, such as HBsAg, HBeAg, and HBV DNA, and serological markers, such as anti-HBs antibodies, anti-HBe antibodies, anti-HBc, and anti-HBc IgM, are beneficial for classifying HBV infection into the various phases of the disease, therapy, and medication response. HBsAg is the gold standard test for diagnosing HBV infection, and it can remain positive for ten weeks after acute exposure. More than six months of HBs Ag persistence suggests chronic HBV infection. Using HBsAg quantification in serum or plasma can evaluate the prognosis of the infection after the clearance of HBsAg in serum and clarify the endemicity of HBV in endemic populations [3, 4, 7, 8, 11, 12].

Anti-HBs in the serum induce long-term immunity in patients. Anti-HB antibodies are the only positive viral marker in the serum of vaccinated individuals. HBeAg is a viral replication and infectivity marker typically positive in the early stages of an infection. Seroconversion of HBeAg to anti-HBe is indicative of hepatic disease remission in viral hepatitis [8, 11].

Anti-HBc indicates exposure to HBV, and in the HBsAg-negative phase of the disease, reactivation of HBV after potent immunosuppression is possible. Anti-HBc is detectable 1 to 2 weeks after the presence of HBsAg, in conjunction with elevated serum aminotransferases and clinical symptoms. Anti-HBc IgM remains positive for six months after acute hepatitis, while only anti-HBc IgG is detectable in the infected patients' serum [7, 10].

In some cases, anti-HBc is a positive marker in patients. This condition includes anti-HBc IgM dominant class during the window period in acute hepatitis, in cases of ended acute infection and undetected serum level of anti-HBs, and long-term chronic HBV infection with undetected HBsAg level [2, 9, 12].

Detection and quantification of HBV DNA via PCR is the most accurate indicator of disease activity in infected patients. This method is a reliable indicator of the replication activity of a virus, as it measures the viral load in patients. The HBV DNA test helps detect and determine antiviral treatment's timing and monitor post-treatment follow-up. Higher HBV DNA titers correlate with hepatocellular carcinoma (HCC) and advanced disease incidence [1–5].

Our evaluation revealed that the patient was positive for HAV IgM, anti-HBc IgM, HBsAg, and HBeAg. Other viral profiles included negative HIV, HDV, and HCV antibody tests. The presence of HAV IgM antibody, HBsAg, and anti-HBc IgM antibodies confirms the patient's diagnosis of acute hepatitis. Whether this is, a coinfection of HBV and HAV or a superinfection of HAV on chronic HBV infection is the primary point of contention. Due to negative previous HBV screening results, absence of high-risk behavior in the previous 11 months, and presence of anti-HBc IgM and HBsAg, acute HBV infection is confirmed; therefore, superinfection of acute HAV on chronic HBV infection is not acceptable for the patient.

Another point of discussion is whether our patient had an acute hepatitis A infection or whether her laboratory result for acute HAV infection was a false positive. Cross-sectional autoantibodies and persistent infections are the primary causes of this issue. Her tests for RF, as well as other viral IgM antibodies (cytomegalovirus (CMV), herpes simplex virus (HSV), and HIV), were negative during our evaluation. Anti-HAV IgG and HAV RNA testing can be utilized to diagnose acute HAV infection. The individual's anti-HAV IgG was negative, but due to limitations in our laboratory testing, we were unable to evaluate HAV RNA. Consequently, acute HAV infection was also confirmed for her based on our evaluation [4–7, 10].

According to the acute hepatitis guidelines, supportive therapy is the only treatment for acute hepatitis infections in healthy individuals with no risk factors. Treatment aims are prevention of acute or subacute liver failure, increased quality of life, and decreased risk of chronicity in patients [7–11].

The presence of coagulopathy (INR >1.5) or prolonged disease (persistent symptoms or stable jaundice for more than four weeks), or signs of acute liver failure, characterize the life-threatening condition of fulminant hepatitis B. According to most cohort studies, early initiation of highly potent antiviral therapy can prevent the development of acute liver failure, liver transplantation, chronicity, and mortality in patients. Some studies have also demonstrated the safety of prescribing tenofovir disoproxil fumarate (TDF), entecavir (ETV), or lamivudine (LAM) for this condition [4, 6, 9, 10, 12].

The patient experienced severe symptoms in our study, including unbearable malaise, generalized jaundice, and persistent nausea and vomiting. The individual had an INR of 1.51, and her aspartate aminotransferase (AST) and alanine aminotransferase (ALT) levels were 2600 U/L and 3590 U/L, respectively. Her alkaline phosphatase (ALP) level was 432 U/L, and her total bilirubin (TB) and direct bilirubin (DB) levels were 15.8 mg/dL and 9 mg/dL, respectively. As a result of her clinical condition and laboratory results, she was admitted and began antiviral treatment.

Our study's strengths were that we promptly identified this rare HAV/HBV coinfection and treated the patient appropriately. Clinical evidence and tests were used to rule out superinfection and confirm a diagnosis of coinfection.

## 4. Conclusion

Hepatitis is a prevalent infectious disease that has been a major global concern. Due to advances in health care and hepatitis vaccination, the risk of these infections has decreased over the past few decades. However, there is always the possibility of hepatitis, especially in endemic areas. In cases where superinfection or coinfection variants of hepatitis are suspected, a complete and detailed history, along with an appropriate virology panel and laboratory tests, can assist the physician in diagnosing the type of hepatitis and prescribing the proper treatment for the patient.

## Figures and Tables

**Table 1:** Laboratory findings during treatment and within one month of follow-up.

Laboratory tests	On admission (before treatment)	3 days after treatment	7 days after treatment	2 weeks after treatment	One month after treatment
AST	2600 U/L	1547 U/L	189 U/L	22 U/L	17 U/L
ALT	3590 U/L	2180 U/L	254 U/L	26 U/L	21 U/L
ALP	432 U/L	337 U/L	328 U/L	298 U/L	287 U/L
Bili.T	15.8 mg/dL	8.3 mg/dL	1.7 mg/dL	1.16 mg/dL	1.1 mg/dL
Bili.D	9 mg/dL	4.5 mg/dL	0.9 mg/dL	0.7 mg/dL	0.5 mg/dL
PT	18	16.2	14	12	12
PTT	41	37	33.5	32	28
INR	1.51	1.4	1.25	1.1	1

AST: aspartate aminotransferase, ALT: alanine aminotransferase, ALP: alkaline phosphatase, Bili.T: total bilirubin, Bili.D: direct bilirubin, PTT: partial thromboplastin time, PT: prothrombin time, INR: international normalized ratio.

## Data Availability

Data and material of this article are not publicly available due to ethical matters but are available from the corresponding author on reasonable requests.
